# Obituary for Guy de Thé

**DOI:** 10.1186/s12977-014-0128-2

**Published:** 2015-01-31

**Authors:** Hugues de Thé, Antoine Gessain

**Affiliations:** Collège de France, INSERM/CNRS U944/7212 and Hôpital St. Louis, Paris, France; Department of virology, CNRS UMR 3569, Institut Pasteur, Paris, France

Guy de Thé, MD, PhD, passed away on August 7, 2014, aged 84. He had obtained his MD in Marseille, as a resident in haematology/Oncology. He then undertook a PhD, which he defended in 1967, partly in France at the Centre National de la Recherche Scientifique (CNRS) in Villejuif, and in the US, working at Duke University with J. Beard and at the National Cancer Institute with R. Bryan. He then became head of the Biological Carcinogene unit at the International Agency for Cancer Research in Lyon up to 1978. He then directed a CNRS laboratory in Lyon and, finally created the Unit of Epidemiology of Oncogenic Viruses at the Institut Pasteur in 1990. After his official retirement in 1997, he actively engaged into academic life, notably at an international level, with the Inter Academy Medical Panel. He was affiliated with the French Academy of Science, of Medicine, the American Institute for Medicine and the Chinese Academy of Preventive Medicine. He has been one of the founders and the President of the International Association for Retrovirology HTLV.

Guy de Thé devoted his research efforts to the oncogenic viruses, particularly in developing countries that pay a high toll to these pathogens. His approaches were particularly broad, ranging from electron microscopy imaging, classic virology, immuno-virology, up to field epidemiology and even anthropology. He trained during all his life, spending multiples stays in the US (Duke, Harvard, NCI), England (Oxford), as well as in China.

**Figure 1:**
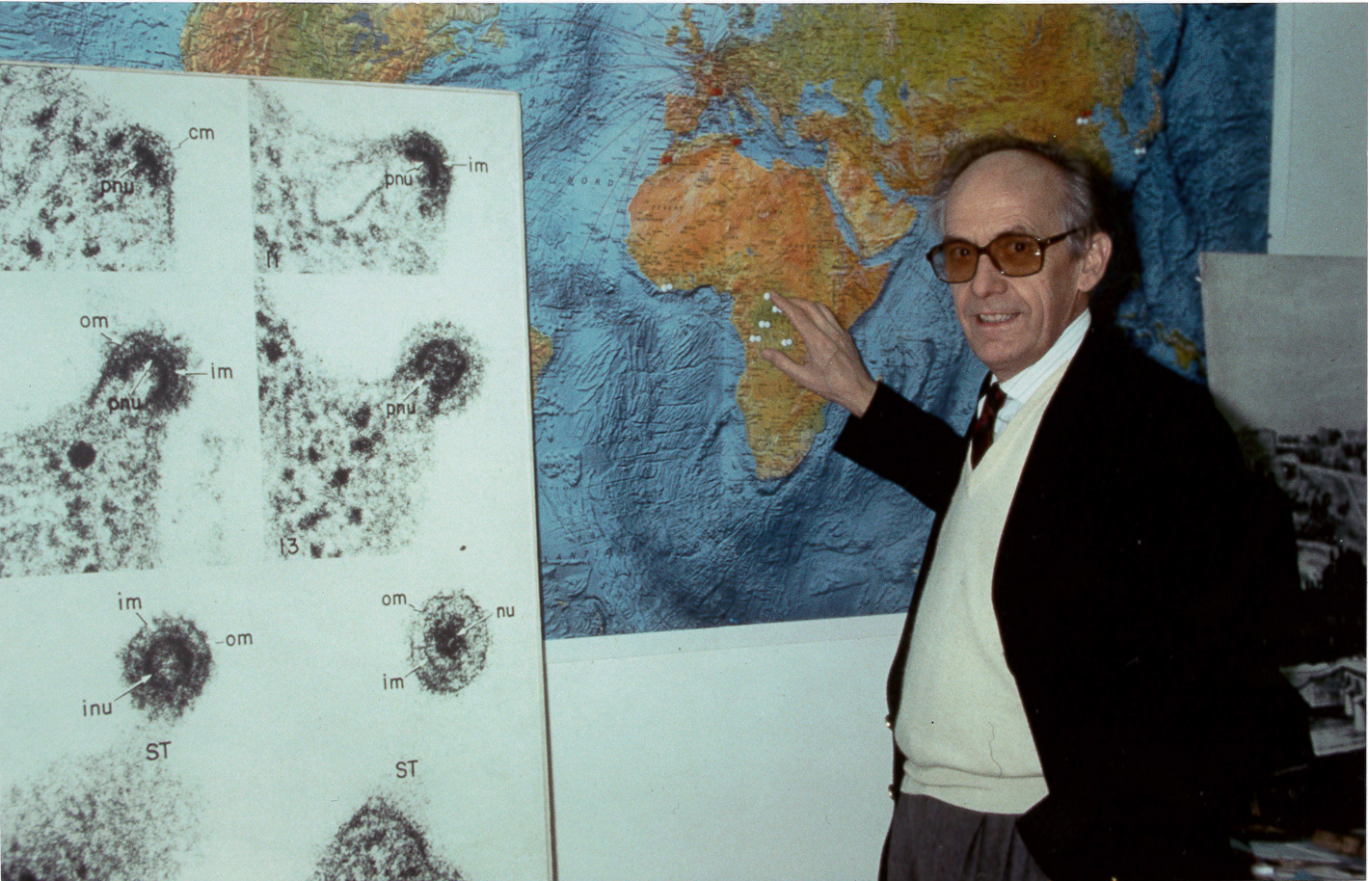
**Guy de Thé in Institut Pasteur Paris in the early 1990’s, showing a map of Africa where the Burkitt prospective study took place.**

Early on, he had chosen viruses, especially herpes-viruses and retroviruses, as the entry point for cancer biology. A passionate advocate for research, ready to take many challenges, he initiated many projects that bridged field and laboratory work, particularly in Africa and Southern China. He was a pioneer in EBV research, prospectively demonstrating, in a very large study performed in Uganda, the key role of early EBV activation in Burkitt's lymphoma [1] (Figure 1). He also pioneered the field of EBV and nasopharyngeal carcinoma (NPC) [2]. Beside some important genetic studies [3], he demonstrated, through large collaboration with Chinese colleagues, that EBV reactivation could be used to predict the development of NPC [4,5]. Finally, while the relationship between retroviral infections and neurodegenerative diseases are well known in animals, he was the first one to demonstrate that HTLV-I infection can drive a chronic and severe neurological condition (Tropical Spastic Paraparesis) frequent in HTLV-1 endemic areas [6,7].

Author of over 300 scientific publications, Guy was also a talented writer, author of many books for the general public in the context of the origin and prevention of cancer. He also organized and was the chairman of several important internationl symposium on mainly oncogenic viruses, especially EBV and HTLV-1.

Guy was an enthusiastic man, always looking for novel clues linking viruses and diseases, anywhere in the world. He had close friends all over the world, notably in China, where he was one of the first French Scientists to establish effective collaborations with Chinese scholars as early as 1977. He trained numerous young researchers and always gave a chance to those who knew how to grab it. His colleagues depict him as a kind, elegant and benevolent man, interested in the discoveries and successes of others. He also had a passion for art, in which he hoped to find the same intrinsic beauties as in biological systems. His friends and family deeply miss him.
